# Site-Specific Variation in Familial Cancer as Suggested by Family History, Multiple Primary Cancer, Age at Onset, and Sex Ratio Associated With Upper, Middle, and Lower Third Esophageal and Gastric Cardia Carcinoma

**DOI:** 10.3389/fonc.2020.579379

**Published:** 2020-10-30

**Authors:** Denggui Wen, Junpeng Wen, Wendi Zou, Yi Yang, Xiaoduo Wen, Yuetong Chen, Kohei Akazawa, Cuizhi Geng, Baoen Shan

**Affiliations:** ^1^ Cancer Center, Hebei Cancer Institute and the Fourth Hospital of Hebei Medical University, Hebei Medical University, Shijiazhuang, China; ^2^ Faculty of Medicine, School of Clinical Medicine, HeBei University, Baoding, China; ^3^ Medical Imaging, Hospital of Sinopec Shengli Oilfield, Dongying, China; ^4^ Department of Medical Image, Hebei Tumor Hospital and the Fourth Hospital of Hebei Medical University, Shijiazhuang, China; ^5^ Department of Medical Information, Affiliated Hospital of Niigata University, Niigata, Japan

**Keywords:** familial cancer, esophageal squamous cell carcinoma, gastric cardia adenocarcinoma, multiple primary cancer, median onset age, family history of cancer

## Abstract

**Background:**

In China, esophageal squamous cell carcinoma (ESCC) and gastric cardia adenocarcinoma (GCA) differ in terms of multiple primary cancer (MPC) and male-to-female sex ratio (MFSR).

**Methods:**

We studied site-specific variation in familial cancer by comparing family history (FH), MPC, age at onset (AO), and MFSR among 8768 patients with ESCC/GCA.

**Results:**

ESCC/GCA patients with a positive FH are associated with a significantly higher rate of MPC and a younger AO than those without (sex-specifically: MPC 1.6% vs. 0.7%, *P*<0.01 and 3.2% vs. 0.8%, *P*<0.01; AO 53.1 ± 8.1 vs. 54.5 ± 8.2, *P*=0.000 and 52.9 ± 7.4 vs. 54.0 ± 8.0, *P*=0.005). Among patients with a positive FH, MPC decreases significantly from upper-, middle-, and lower-third ESCC to GCA (sex-specifically: 53.6%, 1.8%, 1.6%, 0.8%, *P*=0.000; and 71.4%, 1.5%, 2.2%, 1.6%, *P*=0.000). From MPC, upper-, middle-, and lower-third ESCC to GCA, AO increased sex-specifically: 51.9 ± 7.2, 52.8 ± 7.9, 52.1 ± 8.3, 54.3 ± 8.4, 55.6 ± 7.6 (*P*=0.000) and 49.3 ± 6.5, 51.8 ± 9.8, 52.6 ± 7.8, 54.4 ± 8.0, 55.7 ± 7.2 (*P*=0.000), and FH decreased: 43.8%, 35.1%, 28.2%, 29.5%, 24.4% (*P*=0.000) and 55.2%, 26.7%, 25.0%, 24.3%, 22.3% (*P*=0.000). The preponderance of males, smoking, alcohol consumption, and patients ≥50 years old increased from 2.2:1, 1.7:1, 1.0:1, 2.0:1 in ESCC to 6.1:1, 2.8:1, 2.5:1, 4.0:1 in GCA, yet more MPCs were associated with non-preponderant than preponderant counterparts; particularly in GCA, the difference was statistically significant.

**Conclusion:**

The proportion of familial cancer may decrease from upper-, middle-, and lower-third ESCC to GCA. This entails molecular investigation, and appreciating this may help us devise a better screening strategy or individualize cancer treatment.

## Introduction

Worldwide, the highest incidence and mortality rates of esophageal squamous cell carcinoma (ESCC) and gastric cardia adenocarcinoma (GCA) have been found in the north central China endemic region around Linxian (1, 2). These two cancer types are molecularly found to share two susceptibility loci of genome-wide significance: one in PLCE1 at 10q23 and the other in C20orf54 at 20p13 (3, 4). However, tumor biology seems to vary according to the primary site; for instance, upper-third ESCC is more sensitive to radiotherapy than middle- or lower-third ESCC but has the worst prognosis among ESCC patients (5, 6); moreover, although ESCC is associated with more synchronous multiple primary cancer (MPC), GCA exhibits a marked male sex preponderance (7, 8). This study aimed to investigate whether varying proportions of familial cancer are associated site-specifically with ESCC/GCA.

## Materials and Methods

### The High-Risk Region and the Fourth Hospital of Hebei Medical University

The endemic region for ESCC and GCA in north central China (around Linxian) coincides with the Taihang Mountain range, which begins from Henan and Shandong provinces in the southeast and stretches northwestward across Hebei and Shanxi provinces. This constitutes the beginning of the so-called world esophageal cancer belt, which extends westward across most of inland China and several middle-Asian countries as far as Iran. In the early 1950s, a public thoracic cancer center with high-volume surgical capacity was established in each of the provinces of Henan, Hebei, Shanxi, and Shandong to provide surgical treatment for local cases (9). Because ESCC/GCA are prevalent in the endemic region, people complaining of symptoms, such as swallowing disturbance or retrosternal pain, are commonly referred by physicians to the local provincial center. The Fourth Hospital of Hebei Medical University is the cancer center of Hebei Province, where there are approximately 40 million people living in the high-risk region. Most of the 8930 ESCC/GCA patients in the present study were residents of Hebei province. This population constituted almost all consecutive ESCC and/or GCA patients of Hebei province who had undergone thoracic resection with curative intent from 1965 to 1997 because, during that period, the Department of Thoracic Surgery in the Fourth Hospital of Hebei Medical University had been the sole and the most nearby thoracic surgery center specifically established by the government of Hebei province for ESCC/GCA patients. As one of the most prestigious thoracic surgery departments in China, the surgical results were comparable with those in the municipalities of Beijing and Tianjin and the neighboring Henan province (10).

### Patient Enrollment, Data Collection, and Quality Control

**Figure 1:**
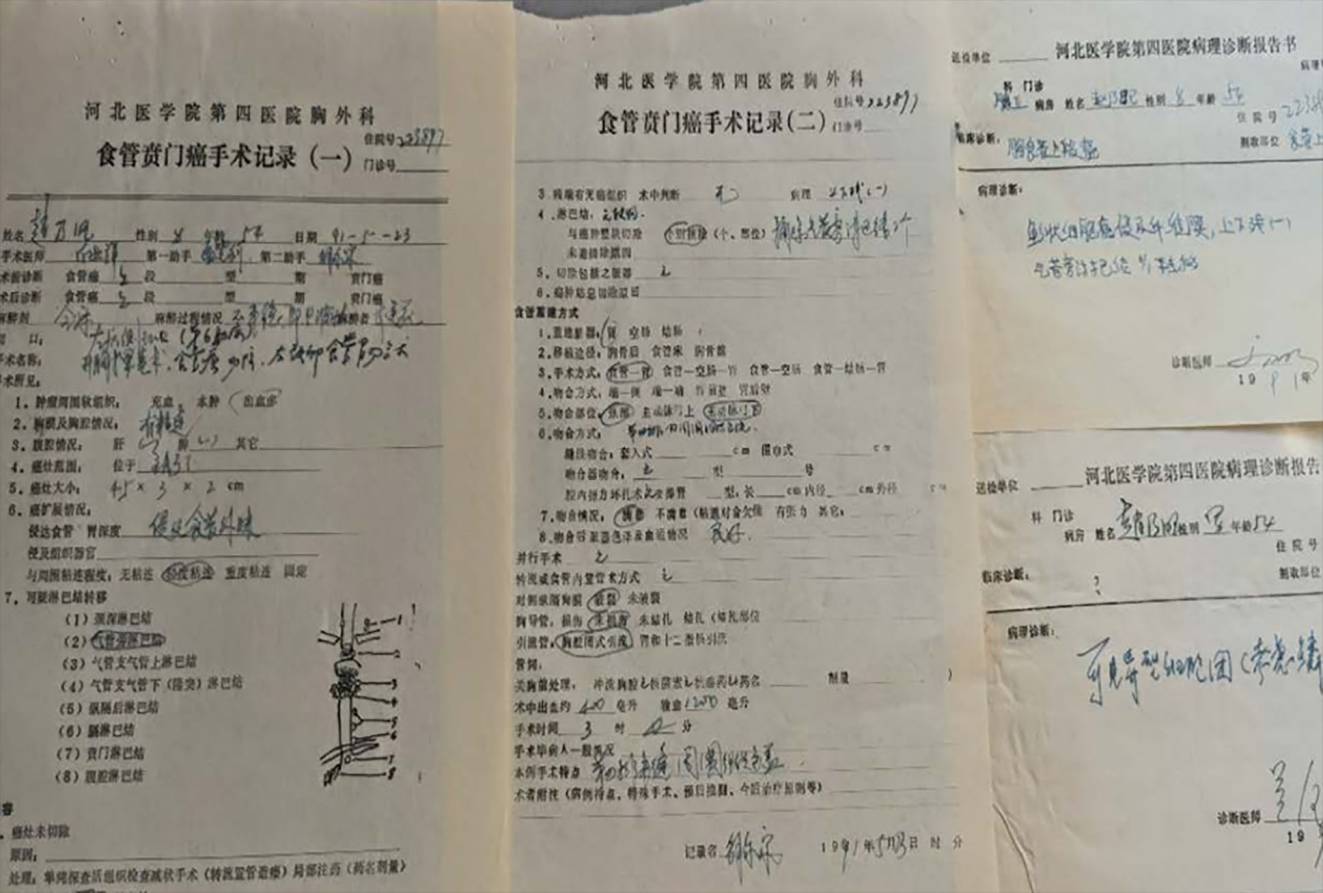
Tumor, nodal, metastasis, and pathological data recorded in the medical chart of patients with esophageal or gastric cardia carcinoma at the Department of Thoracic Surgery in the Fourth Hospital of Hebei Medical University since 1965.

All patients who underwent surgical resection of esophageal and/or gastric cardia carcinoma at the Department of Thoracic Surgery of the Fourth Hospital of Hebei Medical University from 1965 to 1997 were enrolled (*N*=8930). Patients with nonepithelial carcinoma or sarcoma were excluded (*N*=845). Patients who had undergone exploratory surgery or operations for symptom relief only were also excluded because pathological data on tumors, lymph nodes, metastasis, or MPC were incomplete. Although the Department of Thoracic Surgery was established as early as 1952, only patients who underwent resection of ESCC/GCA after 1965 were included because, before that year, data collection or procedures for postoperative histological examination had not been standardized. Since 1965, standard medical charts for surgically treated esophageal or gastric cardia patients have been devised, and clinicopathological data have been collected consistently according to the Western standard ever since (9, 11) (**Figure 1**). The standard and procedures were devised and recommended by the Esophageal Cancer Committee of the China Anticancer Association based on Western experience and were used nationally in major esophageal surgery departments in China (9, 11).

### Diagnosis of Esophageal or Gastric Cardia Cancer

Preoperative diagnoses of esophageal and/or gastric cardia cancer from 1965 to the mid-1980s were established by esophagography, ultrasonography, and balloon cytology as well as later, from the mid-1980s to the late 1990s, by computer-assisted tomography (CT), endoscopy, and biology and, more recently, by ultrasonographic endoscopy or positron emission tomography. Surgery was performed by more than 60 chief thoracic surgeons spanning 3–4 consecutive generations. By 2017, any chief surgeon in the center will have performed a minimum of 400 esophagectomies. Detailed surgical techniques used were introduced by a previous study (10). Detailed pathological data, such as primary tumor site, depth of invasion, histology, grade or differentiation, MPC, nodal involvement, tissue/organ infiltration, and metastasis, were based on pathological examination of the resected specimen and were recorded in the clinical charts (11) (**Figure 1**).

### Data Extraction

Detailed data, including patients’ demographic characteristics, family history (FH), clinical symptoms, diagnostic findings by esophagography, balloon cytology, CT, endoscopy, biopsy, endoscopic ultrasound, and surgical exploration as well as tumor primary site, postoperative pathological examination of depth of tumor invasion, degree of differentiation, lymph node metastasis, distant metastasis, occurrence of surgical complications, and hospital deaths, were all extracted from patients’ charts (11) (**Figure 1**). A data set was constructed using the SPSS (IBM 20.0) data editor. Pathological tumor (T), nodal (N), and metastasis (M) staging was performed specifically for this study by four of the authors (DW, JW, WZ, and YY) according to the UICC/AJCC 7^th^ staging manual based on the postoperative pathological examination data (12).

### Patients’ Follow-Up

For surveillance of surgical results and recurrence, patients are told to visit the surgeon every 3 months during the first 2 years and then every 6 months after discharge. During the visit, esophagography, CT, and laboratory examination are prescribed and recommendations given about rehabilitation. In addition to periodic visits to the clinic, the patient is also contacted every 6 months (by letter or telephone) by a special follow-up group set up by the Department of Thoracic Surgery at the cancer center. This communication with the patient or the family is continued year over year. If response from the patient stops, an active follow-up is made by the follow-up group to the family or to the neighborhood to confirm the survival status. Regarding the survival status of the 8930 patients, by December 31, 2017, 8127 (91%) had died, 536 (6%) were still alive, and 357 (4%) had been lost to follow-up.

### Definition of Age at Onset, FH of Upper Gastrointestinal Tract Cancer, and Synchronous Multiple Primary Esophageal or Gastric Cardia Carcinoma

Age at onset (AO) was determined by the date on which the disease manifested *via* symptoms such as swallowing disturbance or substantial pain. Regarding FH of cancer, the surgeon in charge of the patient asked for detailed information, usually on the first day of hospitalization. A positive FH of upper gastrointestinal tract cancer (UGIC) was defined as (1) at least one first-degree or two second-degree relatives previously diagnosed with UGIC or (2) at least one second- or third-degree relative diagnosed with UGIC at age younger than 50 years or had developed multiple primary UGIC. Relatives of the patients included grandparents, parents and their brothers and sisters, the patient’s siblings, and the patient’s children (fourth generation). Information for FH of cancer includes the site of cancer, blood relationship, time and location of diagnosis, and vital status of the relatives. For a negative FH recalled at the time of hospitalization, the information is continually asked for during the patient’s follow-up. If a first- or second-degree relative of the patient develops UGIC, the FH information is updated. Because ESCC and GCA have similar symptoms, a positive FH usually does not distinguish between the two cancers. Multiple primary esophageal or gastric cardia carcinomas refer to synchronous multiple primary esophageal or gastric cardia lesions discovered during diagnosis or surgery and verified by histological examination. On verification, (1) each primary carcinoma should demonstrate apparent morphologically aggressive characteristics with the multiple primary carcinomas not connected by lymphatic vessels, and (2) each primary carcinoma should be surrounded by intraepithelial neoplasm or by dysplastic lesions (11).

### Data Tabulation and Variables Analyzed

The data of 8930 cases of esophageal and/or gastric cardia carcinoma are classified in **Table 1** by MPC, primary site, and histology. Of these, 162 cases were excluded, including 121 cases of esophageal adenocarcinoma (male, 97; female, 24), 24 cases of esophageal carcinoma other than ESCC or adenocarcinoma (male, 15; female, 9), and 17 cases of gastric cardia carcinoma other than GCA (male, 16; female, 1). The remaining 8768 cases of ESCC/GCA constituted the final data set analyzed and are classified by sex and tumor-site/histology in **Table 2** for comparisons between FH, AO, and male-to-female sex ratio (MFSR). **Figure 1** displays the contrasting age distribution between ESCC and GCA and the preponderance of male cases in GCA.

**Table 1:** Topography and histology of 8930 surgically treated esophageal and/or gastric cardia carcinoma cases at a high-volume center.

Site and histology	Male (*n*=6832)	Female (*n*=2098)	Total (*n*=8930)
Multiple primary carcinoma	64	29	93 (1.0)
Solitary esophageal carcinoma			
Esophageal squamous cell carcinoma	3232 (96.7)	1475 (97.8)	4707 (97.0)
Upper third	74	30	104 (2.2)
Middle third	2365	1070	3435(73.0)
Lower third	793	375	1168(24.8)
Esophageal adenocarcinomas	97 (2.9)	24 (1.6)	121 (2.5)
Upper and middle third	34	11	45(37.2)
Lower third	63	13	76(62.8)
Esophageal cancer: other types	15 (0.4)	9 (0.6)	24 (0.5)
Total solitary esophageal carcinomas	3344	1508	4852 (54.4)
Solitary gastric cardia carcinoma			
Gastric cardia adenocarcinoma	3408 (99.5)	560 (99.8)	3968 (99.6)
Gastric cardia cancer: other types	16 (0.5)	1 (0.2)	17 (0.40)
Total of solitary gastric cardia carcinoma	3424	561	3985 (44.6)
Total (%)	6832 (76.5)	2098 (23.5)	8930 (100.00)

**Table 2:** Decreasing family history, increasing median age at onset, and male/female sex ratio associated with 8768 patients^a^ with esophageal squamous cell carcinoma and/or gastric cardia adenocarcinoma according to multiplicity, tumor site, and histology.

Multiplicity, site, and morphology	Male (*n*=6704)			Female (*n*=2064)			Male/Female ratio
	*n*	Family history of UGIC, *n* (%)	Mean onset, age ± SD	*n*	Family history of UGIC, *n* (%)	Mean onset, age ± SD	
Multiple primary UGIC cases	64	28 (43.8)	51.9 ± 7.2	29	16 (55.2)	49.3 ± 6.5	2.2
Positive FH	28	28 (100.0)	49.8 ± 5.1	16	16 (100.0)	45.9 ± 3.8	1.8
Negative family history	36	0 (0)	53.6 ± 8.2	13	0 (0)	53.6 ± 6.6	2.8
*P*			0.038^d^			0.001^d^	0.372^b^
Solitary esophageal squamous carcinoma	3232			1475			2.2
Upper third	74	26 (35.1)	52.8 ± 7.9	30	8 (26.7)	51.8 ± 9.8	
Middle third	2365	653 (28.2)	52.1 ± 8.3	1070	267 (25.0)	52.6 ± 7.8	
Lower third	793	249 (29.5)	54.3 ± 8.4	375	91 (24.3)	54.4 ± 8.0	
Gastric cardia adenocarcinoma	3408	830 (24.4)	55.6 ± 7.6	560	125 (22.3)	55.7 ± 7.2	6.1
*P*		0.000^b^	0.000^d^		0.003^b^	0.000^d^	0.000^b^
*P*		0.000^c^	0.000^e^		0.041^c^	0.000^e^	
Total	6704	1786 (26.6)	54.2 ± 8.2	2064	507 (24.6)	53.8 ± 7.9	3.3

^a^A total of 162 cases were excluded from 8930 cases as listed in **Table 1**, including 121 cases of esophageal adenocarcinoma (male 97, female 24), 24 cases of esophageal carcinoma other than ESCC (male 15, female 9), and 17 cases of gastric cardia carcinoma other than GCA (male 16, female 1).

^b^P value for difference between groups by Pearson chi-square test; ^c^P value for linear-by-linear association by chi-square test; ^d^P value for difference between groups by ANOVA; ^e^P value for the linear term by ANOVA.

Although the rate of synchronous MPC may indicate the proportions of familial cancer (13), it would be either 100% with the multiple primary cases or zero with the solitary carcinoma cases if classified as in **Table 2**. Therefore, the 93 MPC cases are subdivided into 177 solitary lesions of ESCC/GCA; thus, together with the 8675 ESCC/GCA developed by the 8675 single-lesion patients, a total of 8852 lesions are reclassified in **Table 3** according to site/histology, sex, and FH for comparison of the rate of MPC.

**Table 3:** Proportion of MPC associated with 8852^a^ separate ESCC and/or GCA by tumor site, sex, and family history of upper gastrointestinal cancer.

Tumor site/histology	Male (*n*=6763)				Female (*n*=2089)			
	Positive FH		Negative family history		Positive FH		Negative family history	
	*n*	MPC (%)	*n*	MPC (%)	*n*	MPC (%)	*n*	MPC (%)
Upper third ESCC	56	53.6	48	0.0	28	71.4	22	0.0
Middle third ESCC	666	1.8	1707	2.7	271	1.5	825	2.7
Lower third ESCC	252	1.6	601	1.0	93	2.2	285	0.4
GCA	837	0.8	2596	0.7	127	1.6	438	0.7
*P*		0.000^b^		0.000^b^		0.000^b^		0.011^b^
*P*		0.000^c^		0.000^c^		0.000^c^		0.008^c^

^a^Including 8675 ESCC/GCA developed by the 8675 solitary tumor cases and 177 ESCC/GCA developed by the 93 multiple tumor cases.

^b^P value for difference between groups by Pearson chi-square test; ^c^P value for linear-by-linear association by chi-square test.

### Statistical Methods

The SPSS statistical package, version 20.0 for Windows (IBM, Armonk, NY, USA) was used to construct data sets and perform statistical analyses. The chi-square test was used to test the difference in proportions of sex, age, smoking, alcohol consumption, and MPC between patients with ESCC or GCA, and the Mann–Whitney U-test was employed to test the stage-specific difference in age between ESCC and GCA. For statistical comparison according to the upper-, middle-, and lower-third ESCC and GCA, one-way analysis of variance (ANOVA) was employed to test the difference in age and the linear trend, and the Pearson chi-square test was used to examine the difference in proportions of FH and to test the linear-by-linear association. All *P* values were two-tailed, and the level of *P*<0.05 was considered statistically significant.

The study was approved by the institutional ethics review board of the Fourth Hospital of Hebei Medical University (ID 20190030).

## Results

### Topography and Histology

Of the 8930 patients with esophageal or gastric cardia carcinoma, 4852 (54.4%) developed a single primary esophageal carcinoma, 3985 (44.6%) developed a solitary gastric cardia carcinoma, and 93 (1.0%) developed multiple primary esophageal or gastric lesions (**Table 1**).

Among esophageal carcinomas, 97.0% (4707/4852) were squamous cell carcinomas, 2.5% (121/4852) were adenocarcinomas, and 0.5% (24/4852) were of other histological types. In subsite distribution, 2.2%, 73.0%, and 24.8% of ESCC were located at the upper-, middle-, and lower-third of the esophagus, and 37.2% and 62.8% of the esophageal adenocarcinomas were located at the top two thirds and the lower third, respectively.

Regarding the histology of gastric cardia carcinoma, 99.6% (3968/3985) were adenocarcinomas, and 0.51% (17/3985) were other cancer types.

Concerning the 93 multiple primary UGIC cases, three developed triple-primary and 90 developed double-primary UGIC. The three triple-primary patients each had two ESCC and one GCA. Among the 90 double-primary patients, 28 had both a middle-third ESCC and a GCA, 14 had both a lower-third ESCC and a GCA, one had both a middle-third ESCC and a middle-third esophageal adenocarcinoma, 36 had two primary ESCC, three had both an esophageal adenocarcinoma and a GCA, six had both an ESCC and a primary distant gastric carcinoma, and two had both a GCA and a distant gastric adenocarcinoma. Overall, a total of 177 ESCC/GCA, four esophageal adenocarcinomas, and eight distant gastric carcinomas were developed by the 93 multiple primary UGIC cases. Calculation of the rate of MPC was based on the 177 ESCC/GCA with the four esophageal adenocarcinomas and eight distant gastric tumors excluded.

### Decreasing FH Associated With Patients With Multiple Primary; Upper-, Middle-, and Lower-Third ESCC; and GCA

Of the 8768 patients with ESCC/GCA, 2293 (26.2%) had recalled a positive FH of UGIC. Among these, 79.4% involved at least one first-degree relative and 20.6% involved at least two second-degree relatives. For both sexes, a positive FH was most frequently recalled by the MPC cases (43.8% and 55.2%), and for patients with upper-, middle-, and lower-third ESCC and GCA, the percentage was 35.1%, 28.2%, 29.5%, 24.4% and 26.7%, 25.0%, 24.3%, and 22.3% sex-specifically (**Table 2**). Overall, FH showed a significantly downward trend from MPC to upper-, middle-, and lower-third ESCC to GCA in both male (*P* =0.000) and female (*P* =0.000) patients.

### AO Increased Site-Specifically for Patients With Multiple Primary Tumor; Upper-, Middle-, and Lower-Third ESCC; and GCA

In **Table 2**, AO associated with patients with MPC; upper-, middle-, and lower-third ESCC; and GCA increased significantly by 51.9, 52.8, 52.1, 54.3, and 55.6 (*P *=0.000) and 49.3, 51.8, 52.6, 54.4, and 55.7 years old (*P *=0.000). As shown in **Figure 1**, there were significantly more cases with younger AO in ESCC than in GCA irrespective of sex.

### Male-to-Female Sex Ratio Increased Significantly From ESCC to GCA

**Figure 2:**
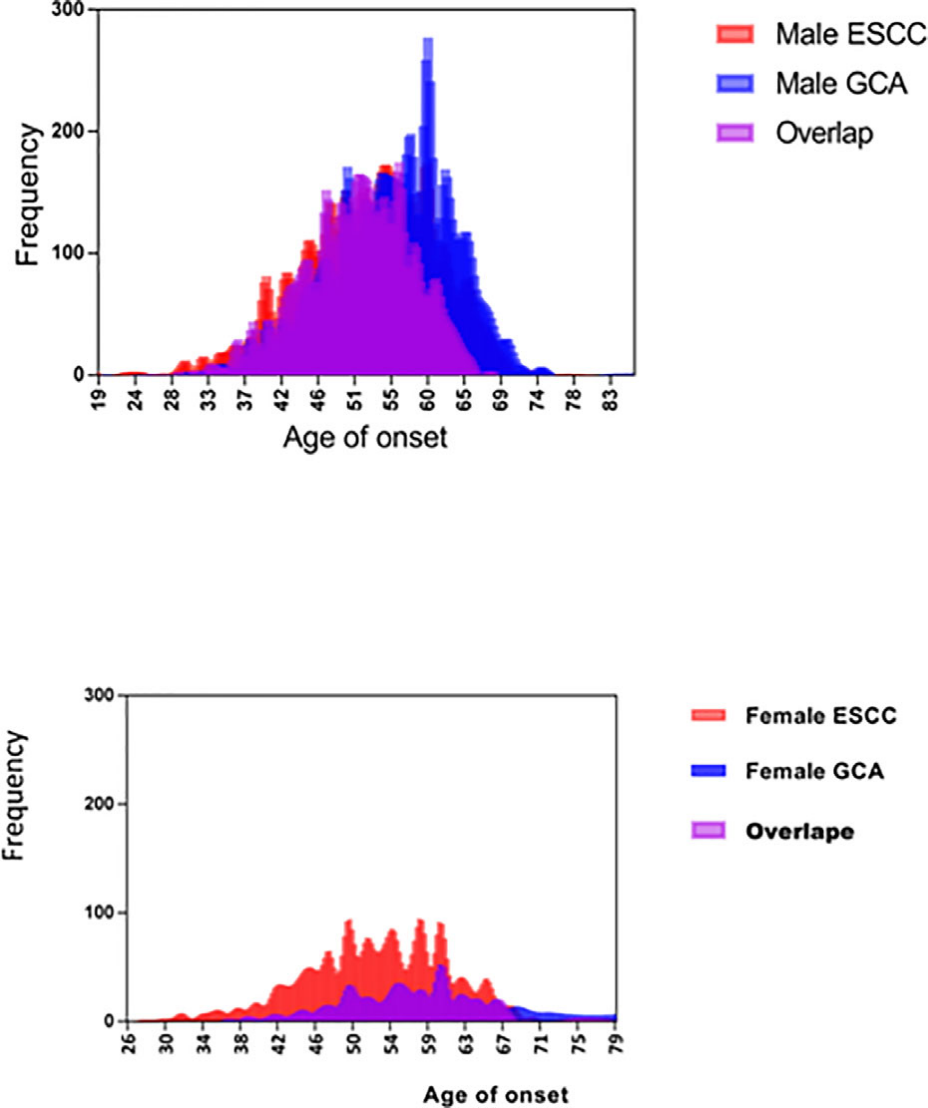
Increased preponderance of male sex and older age at onset associated with patients with GCA in comparison with patients with ESCC.

As shown in **Table 2**, the MFSR associated with ESCC was significantly larger than that associated with GCA (2.2:1 vs. 6.1:1, χ^2^ = 10.6, *P <*0.001). In **Figure 2**, GCA is associated with a marked male sex preponderance in comparison with ESCC (*P*<0.01).

### Association Between FH, Multiple Primary Tumor, and AO

Overall, ESCC/GCA patients with a positive FH of UGIC were associated with a significantly higher rate of MPC and a younger AO than those without (MPC in males 1.6% vs. 0.7%, *P*<0.01; MPC in females 3.2% vs. 0.8%, *P*<0.01; AO of males 53.1 ± 8.1 vs. 54.5 ± 8.2 years old, *P*=0.000; AO of females 52.9 ± 7.4 vs. 54.0 ± 8.0 years old, *P*=0.005). Among patients with MPC, the AO of patients with a positive FH was significantly younger than those without between both males (49.8 ± 5.1 vs. 53.6 ± 8.2 years old, *P*=0.038) and females (45.9 ± 3.8 vs. 53.6 ± 6.6 years old, *P*=0.001) (**Table 2**).

### Decreasing MPC Associated With Upper-, Middle-, and Lower-Third ESCC and GCA

As shown in **Table 3**, among lesions developed by patients with a positive FH of UGIC, the rate of MPC decreased significantly from the upper-, middle-, and lower-third ESCC to GCA in both males (53.6%, 1.8%, 1.6%, 0.8%; *P*=0.000) and females (71.4%, 1.5%, 2.2%, and 1.6%; *P*=0.000). Among lesions developed by patients without an FH, although no MPC were associated with upper-third ESCC, the rate also decreased significantly from the middle- and lower-third ESCC to GCA in both males (2.7%, 1.0%, 0.7%; *P*=0.000) and females (2.7%, 0.4%, 0.7%; *P*=0.008).

### Preponderance of Male Sex, Smoking, Drinking, or Older Onset Age Increased From Patients With ESCC to Patients With GCA, Yet More MPC Were Associated With Non-preponderant Than Preponderant Cases

As shown in **Table 4**, a marked preponderance of male sex, smoking, alcohol consumption, and patients older than 50 years was observed in GCA as compared with ESCC, yet more MPC were associated with the non-preponderant than the preponderant counterparts, and the difference was significant in GCA but not in ESCC cases. This increased MPC may indicate a selection for genetic susceptibility in patients less exposed to environmental risk.

**Table 4:** Increased preponderance of males, smoking, drinking, and older cases associated with GCA compared with ESCC and difference in MPC (%) between preponderance and non-preponderance.

Patients’ characteristics	Ratio		Ratio difference	Difference in MPC (%) between preponderance and non-preponderance, and *P* value			
	ESCC	GCA	*P* ^a^	ESCC (% vs %)	*P* ^a^	GCA (% vs %)	*P* ^a^
Male vs. female	2.2:1	6.0:1	0.01	2.4% vs. 2.8%	0.35	1.3% vs. 3.0%	<0.01
Smoker vs. nonsmoker	1.7:1	2.8:1	0.01	2.6% vs. 3.0%	0.14	1.4% vs. 2.4%	<0.05
Drinker vs. nondrinker	1.0:1	2.5:1	0.01	2.3% vs. 2.8%	0.40	1.1% vs. 2.3%	<0.05
≥50 vs. <50 years old	2.0:1	4.0:1	0.01	2.7% vs. 2.2%	0.20	1.2% vs. 2.7%	<0.01

^a^P values by Pearson chi-square test.

## Discussion

Tumors develop by interaction between inherited susceptibility and accumulation of somatic mutations in later life. In this sense, they may be accordingly divided approximately into inherited cancer syndrome and familial or sporadic cancer types. If a person is genetically susceptible, few environmental mutations may be needed, so a familial cancer may be diagnosed at an earlier age than a sporadic cancer. A positive FH of related cancer is more likely associated with a familial than a sporadic cancer. Because inherited predisposition, such as a mutation in one allele of a tumor-suppressor gene, theoretically exists in every cell, against such a genetic background, familial cancers are likely to develop at multiple sites, whereas most sporadic cancers develop as a solitary lesion. Therefore, MPC may serve as another indicator for genetic predisposition (13).

In 2009, when analyzing the same population but with a smaller surgical cohort (operated on from 1985 to 1994), we found that ESCC cases with a positive FH of UGIC (*n*=477) are more likely to develop MPC and have a poorer survival than those without such a history (*n*=1238) (7). In 2013, upon finding systematic differences in FH of UGIC, multiple primary lesions, median AO, and MFSR between 3711 cases of ESCC and 3310 cases of GCA surgically resected between 1970 and 1994, we postulated that ESCC may embrace more familial cancer than GCA (8). This time, we recruited all ESCC and GCA cases resected at the Fourth Hospital of Hebei Medical University between 1965 and 1997, during which time the hospital had been the sole thoracic surgery center of Hebei Province. Here, we found that the percentage of FH of UGIC and the rate of synchronous MPC decreased significantly, and the median age at diagnosis and MFSR increased significantly according to upper-, middle-, and lower-third ESCC and GCA. These observations suggest that a decreasing proportion of familial cancer may be associated with UGIC from top to bottom in this endemic region. Considering that molecular investigation of genetic susceptibility is possible only through studying familial cancer cases, population-based cancer control programs should focus more on the elimination of environmental hazards to prevent sporadic cancer cases while understanding that the proportion of familial/sporadic cancer is an imperative.

Clinicopathological studies carried out worldwide on esophageal cancer show some agreement with the present findings. Piessen et al. argue that cervical and upper-third thoracic esophageal carcinoma has distinct clinical features and should be regarded as a single pathological entity (5). Regarding the prognosis of esophageal cancer, Sorrentino notes that the higher the position of the tumor, the worse the prognosis (6). Papp et al. once compared the effect of 4-week-long preoperative chemoradiotherapy between 40 cases of upper-third ESCC and 62 cases of middle-third ESCC. In the end, complete remission was achieved in 35% (14/40) of the former group but in only 4.8% (3/62) of the latter group (*P <*0.05), suggesting a site-specifically different tumor biology (14).

Other studies report further that what upper aerodigestive cancers share in genetic predisposition may vary in terms of the cancer site (15, 16). A single-nucleotide polymorphism in PLCE-1, for instance, has been consistently found to be associated with the risk of ESCC, GCA, and head and neck cancer in the Chinese population (3, 4, 17). With head and neck cancer, effects of risk alleles of PLCE-1 were associated with cancers of the oral cavity, hypopharynx, or larynx but not with cancers of the oropharynx (17). Lee et al. report the results of a population-based study regarding the risk of developing a second primary esophageal cancer in 33,787 patients with oral and pharyngeal carcinoma over a 25-year period, whereby the standardized incidence ratios increased as the site of oral and pharyngeal carcinoma decreased, for example, from 5.57 (95% confidence interval [CI] 4.53–6.78) for carcinoma at the oral cavity to 14.29 (95% CI 9.64–20.39) at the oropharynx to 22.76 (95% CI 17.77–28.70) at the hypopharynx, suggesting site-specific inherited susceptibility (18).

In addition to a positive FH, early age at diagnosis, and multiple primary lesions, tumors under the influence of an inherited susceptibility occasionally affect young females and are usually associated with a small sex ratio as exemplified by the contrast between the diffuse and intestinal types of gastric adenocarcinoma in the Lauren classification (19). Similarly, in **Table 2**, multiple primary cancer cases and ESCC have significantly lower MFSRs than GCA (2.2:1 vs. 2.2:1 vs. 6.1:1). Dawsey et al. report on 60 patients diagnosed with ESCC by the age of 30 years or younger in Africa, in whom the male/female ratio was only 1.4:1 (35:25). Female patients rarely smoked or drank in comparison with males (smoking 0% vs. 26%; drinking 4% vs. 23%), but positive FH of esophageal cancer was recalled more frequently by female than by male patients (57% vs. 32%) (20).

Alternatively, if the overwhelming majority of cancer cases is determined by environmental or lifestyle hazards, for the environmentally less exposed, such as female, nonsmoking, or nondrinking patients, only those carrying susceptible genotypes may compensate for less environmental exposure (21); therefore, although the number of such cases is few under selection, they tend to develop MPC. This may be the reason for the marked preponderance of male, smoking, drinking, or older cases as well as the more frequent development of MPC in the non-preponderant than their preponderant counterparts in GCA as compared with ESCC (**Table 4**).

The following arguments may be offered to strengthen the present findings. First, although excessive smoking and drinking are widely acknowledged as risk factors for MPC arising in the upper aerodigestive tract (22), in the present study, relatively more MPC developed among nonsmoking, nondrinking, and female patients (**Table 4**). In addition, smoking or drinking is not related to cancer-site distribution in the present study (percentage of smokers among upper-, middle-, and lower-third ESCC and GCA was 70%, 82%, 77%, and 76% in males [*P*=0.45] and 8%, 13%, 9%, and 9% in females [*P*=0.64]; percentage of alcohol drinkers was 41%, 56%, 56%, and 55% in males [*P*=0.47] and 4%, 4%, 4%, and 4% in females [*P*=0.82]). Second, earlier dysphagia for ESCC than for GCA may be due to anatomical reasons rather than cancer development. However, with each pTNM stage (UICC/AJCC 7^th^ edition) (12), ESCC is diagnosed at a younger age than GCA, and the discrepancy is larger at early than at late stages (**Table 5**). Third, MPC is rarer in the gastric cardia than the esophagus, perhaps because, anatomically, the former is only 2–3 cm wide across the most proximal stomach. However, data in **Table 4** prove this suspicion unlikely because MPC are more frequently found in female or younger-onset GCA patients than their corresponding ESCC counterparts. Fourth, a single-center sample may not be representative of the general patient population. However, before 1998, Hebei province had the sole thoracic surgery center in this hospital. The hospital (Shijiazhuang city) is located within the endemic area. In fact, the hospital had been set up especially by the government in 1952 to provide thoracic surgery for the endemic area of Hebei province, and the department has continued to be one of the pioneering departments in China. From 1952 to 1988, 9601 patients had undergone resection at the hospital with an annual volume of 260 cases. From 1990 to 2000, 6894 patients underwent surgery, and the annual capacity reached more than 600 (9). Because thoracotomy and esophagogastrostomy requires expertise and is resource demanding, most ESCC/GCA cases in the province were referred to this center although some exceptions might exist. Epidemiological data also suggest that the present sample is representative of the general patient population; ESCC or GCA represented 53.95% and 46.05% in the present series, and in the Linxian general population study, ESCC and GCA constituted 57.4% and 31.9% of gastroesophageal cancers (1). The values for median AO, MFSR, and percent FH of UGIC in the present study are also similar to those reported in the Linxian general population study (1) and another report carried out in China (23).

**Table 5:** Difference in median age at onset between ESCC and GCA according to tumor stage (UICC/AJCC 7th edition).

Sex	Stage^a^	AO		Difference in AO	*P* value^b^
		ESCC	GCA		
Male	T is, 1 N0M0	55.0 ± 9.5	60.0 ± 8.2	–5.0	<0.002
	T 2, 3 N0M0	53.0 ± 8.4	57.0 ± 8.0	–4.0	<0.0001
	T 2, 3, 4 N1M0	54.0 ± 8.4	56.0 ± 8.6	–2.0	<0.0001
	T 2, 3, 4 N2M0	52.0 ± 8.0	55.0 ± 8.4	–3.0	<0.0001
	T 2, 3, 4 N3M0	54.0 ± 8.9	59.5 ± 8.7	–5.5	<0.001
	T 2, 3, 4 N3M1	54.5 ± 8.7	59.5 ± 8.4	–5.0	<0.001
	Total	53.0 ± 8.5	56.0 ± 8.5	–3.0	<0.0001
Female	T is, 1 N0M0	55.0 ± 5.4	60.0 ± 8.2	–5.0	0.10
	T 2, 3 N0M0	53.0 ± 8.3	57.0 ± 8.4	–4.0	<0.0001
	T 2, 3, 4 N1M0	54.0 ± 8.5	56.0 ± 8.1	–2.0	<0.01
	T 2, 3, 4 N2M0	54.7 ± 8.4	55.9 ± 8.0	–1.2	<0.01
	T 2, 3, 4 N3M0	55.0 ± 9.0	55.9 ± 8.2	–0.9	0.16
	Total	53.0 ± 8.5	56.0 ± 8.3	–3.0	<0.0001

^a^T is, 1 N0M0 means the T stage is either carcinoma in situ or in stage 1, the N stage is 0, and the M stage is 0.

^b^P values by Mann–Whitney U-test.

In conclusion, decreasing MPC and FH of UGIC and increasing mean AO and MFSR according to upper-, middle-, and lower-third ESCC and GCA suggest that the proportion of familial cancer among UGIC may decrease site-specifically. Appreciating this may help us to devise a better screening strategy or individualize cancer treatment. Etiologically it is worthwhile investigating the underlying mechanism although the present results need to be confirmed by larger population-based studies.

## Data Availability Statement

The raw data supporting the conclusions of this article will be made available by the authors upon request, without undue reservation.

## Ethics Statement

The studies involving human participants were reviewed and approved by The study was approved by the Institutional Ethics Review Board of 4th Hospital of Hebei Medical University. The patients/participants provided their written informed consent to participate in this study.

## Author Contributions

All authors contributed to the article and approved the submitted version. DW, JW, WZ, KA, CG, and BS led the study design. DW, JW, WZ, YY, XW, YC, and CG abstracted medical charts, checked original diagnoses, staged tumors, constructed data sets, performed statistical analyses, and drafted the manuscript. DW, JW, KA, and BS contributed to writing and revising the manuscript.

## Funding

This work was partially supported by a grant for Key subject development for universities of Hebei Province (No. 03276198D).

## Conflict of Interest

The authors declare that the research was conducted in the absence of any commercial or financial relationships that could be construed as a potential conflict of interest.
